# Continuous input drives motor cortical dynamics during reaching

**DOI:** 10.21203/rs.3.rs-8585660/v1

**Published:** 2026-02-12

**Authors:** Hongwei Mao, Brady A. Hasse, Andrew B. Schwartz

**Affiliations:** 1. Department of Neurobiology, University of Pittsburgh School of Medicine, Pittsburgh, PA 15261, USA; 2. Systems Neuroscience Center, University of Pittsburgh School of Medicine, Pittsburgh, PA 15261, USA; 3. Department of Bioengineering, University of Pittsburgh Swanson School of Engineering, Pittsburgh, PA 15261, USA

## Abstract

In a departure from current models of autonomous cortical activity, we examined the effect of continuous input on the dynamic activity of motor cortical neurons throughout a reaching task. We found a series of distinct state transitions in the firing rates of these recorded neural populations. We then asked whether these changes in state were due to internal processing in the motor cortex, as previous models would suggest, or to continuous external inputs. In other words, were signals coming from outside the motor cortex the main drivers of neural activity during reaching behavior? To answer this question, we used hybrid neural networks (HNNs)—consisting of a mixture of artificial connectivity and empirical firing rates—to construct realistic model systems. The HNNs faithfully produced the firing rates of the individual neurons and the state transitions of the populations we recorded, with extrinsic input consisting of episodically modulated neurons. Instead of the reported primacy of intrinsic action, we found that input from extrinsic sources was responsible for these results. Episodic external drive produced consistent changes in the statistics of pre-threshold input integration to cause the state transitions. By using HNNs with empirically constrained connectivity, we have shown that continuous input is a plausible agent for broad system functionality.

## Introduction

Volitional movement allows us to act on the world^1^. But despite its importance, there is no consensus on how neural processing generates intentional physical action. A cornerstone concept is the classic notion of a “motor program,”^2–5^ defined as a “set of muscle commands that are structured before a movement sequence begins that allows the entire sequence to be carried out uninfluenced by peripheral feedback”^2^. The basic idea is that, before any movement, the nervous system selects a template from a stored library (memory) of instructions (muscle commands) that dictates the subsequent action. This concept has evolved over the last 50 years^6,7^, with shifting ideas about the nature of the stored motor command. The original premise of a complete prespecified plan was modified in a subsequent definition to include “… appropriate compensatory adjustments as the action unfolds”^7^, suggesting a framework in which ongoing control takes place throughout the movement. More specifically, this modified definition only provides a substrate for the generation of motor commands without representing the commands themselves. Subsequent challenges to the original pre-specification criterion came in the early ‘90s, when neurophysiologists found this aspect of programming to be vague and implausible as a model of the motor system^8^. A dynamical systems approach coming from psychophysics^9,10^ further challenged the original pre-specification tenet by arguing that physical structure governs the way movements are formed and controlled. While retaining the autonomous aspect of motor programs, this dynamical systems argument found that memory storage wasn’t needed, as directed movement could be “self-assembled”^9^.

In recent years, a similar dynamical systems model has been applied to neural activity recorded from motor cortical areas during primate arm movement^11^. This model conforms to the earlier, behaviorally derived dynamical systems theory, with the idea that neural activity sets an initial condition that reflects the intention to make a particular movement—an example of self-assembly. According to this scheme, activity of interconnected cortical neurons migrates toward a network state to initiate a specific “neural trajectory”; this trajectory in turn autonomously produces a set of muscle contractions to move the arm. Both the old and new versions of dynamical systems maintain that a complete set of commands is determined before a volitional movement begins as a form of set-and-forget control.

In contrast, other behavioral studies of reaching emphasize continuous control^12^. The concept of autonomous motor control runs counter to schemes that stress the importance of feedback throughout movement^13–18^. Perhaps the most compelling argument for ongoing control is the success of brain-computer interfaces (BCIs) that restore function to paralyzed individuals by decoding a robust stream of instantaneous information from motor cortical activity throughout an intended action^19–28^. Yet, while the dynamic details of movement are decoded continuously in this paradigm, it remains an open question whether this information is derived from the autonomous unwrapping of a static plan or from a continuous process using feedback (e.g., vision of the decoded movement).

There is no question that behavior generation is based on prediction^1^. At a high level, theories of mind postulate that actions are chosen by learning their consequences on the world^29^. At an intermediate level, there is an obligatory reaction time of about 300 ms between target presentation and the onset of a reach^30^. Lower still, dynamic population vectors calculated from motor cortical activity precede the corresponding velocity vector of the hand’s trajectory by approximately 120 ms^20,21^. With respect to motor cortical activity in the motor cortex, set-and-forget control would have a prediction interval of 300–500 ms (reach duration), in contrast to the approximate 100 ms interval for continuous control during the movement.

In this paper, we extend the current dynamical systems approach by considering behaviorally linked control. Kelso, in his original essays on dynamical systems applied to motor behavior^9^, described the systems as dissipative, non-equilibrium, non-linear, and densely interconnected, with different states between phase transitions recognizable by their patterns of organization. We found a sequence of patterns in the firing rates recorded from populations of motor cortical neurons during reaching. The same neurons participated in each state, another characteristic of the original dynamical systems formulation. While these patterns confirm one aspect of Kelso’s theory, they are driven by ongoing extrinsic input, which does not conform to his concept of predetermination.

Sequential states with multi-functional neurons^31^ are inherent in artificial neural networks, which can be used as model dynamical systems^32–36^. We built hybrid neural network models (HNNs) with dynamic input to replicate our empirical data. The HNN models show how extrinsic input, modulated in concordance with external events, can drive the state transitions that take place throughout a single movement. Distinct patterns of modeled synaptic integration within each state show how disparate input to individual neurons can be combined to affect a neuron’s functionality. This modeling, constrained by empirical data, provides a mechanistic explanation for functional connectivity. The models show how dynamical systems, operating through a series of control processes, participate in the generation of volitional movement. These findings emphasize continuous control of movement and show that behavioral output is unlikely to be completely predetermined.

## Results

### Empirical Data

We recorded simultaneous single-unit activity using Utah arrays in two monkeys (Fig. S1) as they performed a center-out reach task (Fig. S2) while viewing a virtual reality monitor (Methods). Simultaneous recordings were made from 67 units in the primary motor cortex of Monkey C and from 78 units in the dorsal premotor cortex of Monkey N. Three-dimensional hand position data from Monkey C were recorded at 60 Hz, and from Monkey N at 100 Hz. Together, these form the database for this study (Methods).

### Discrete epochs in firing rates of individual neurons during reach

**Figure 1.**
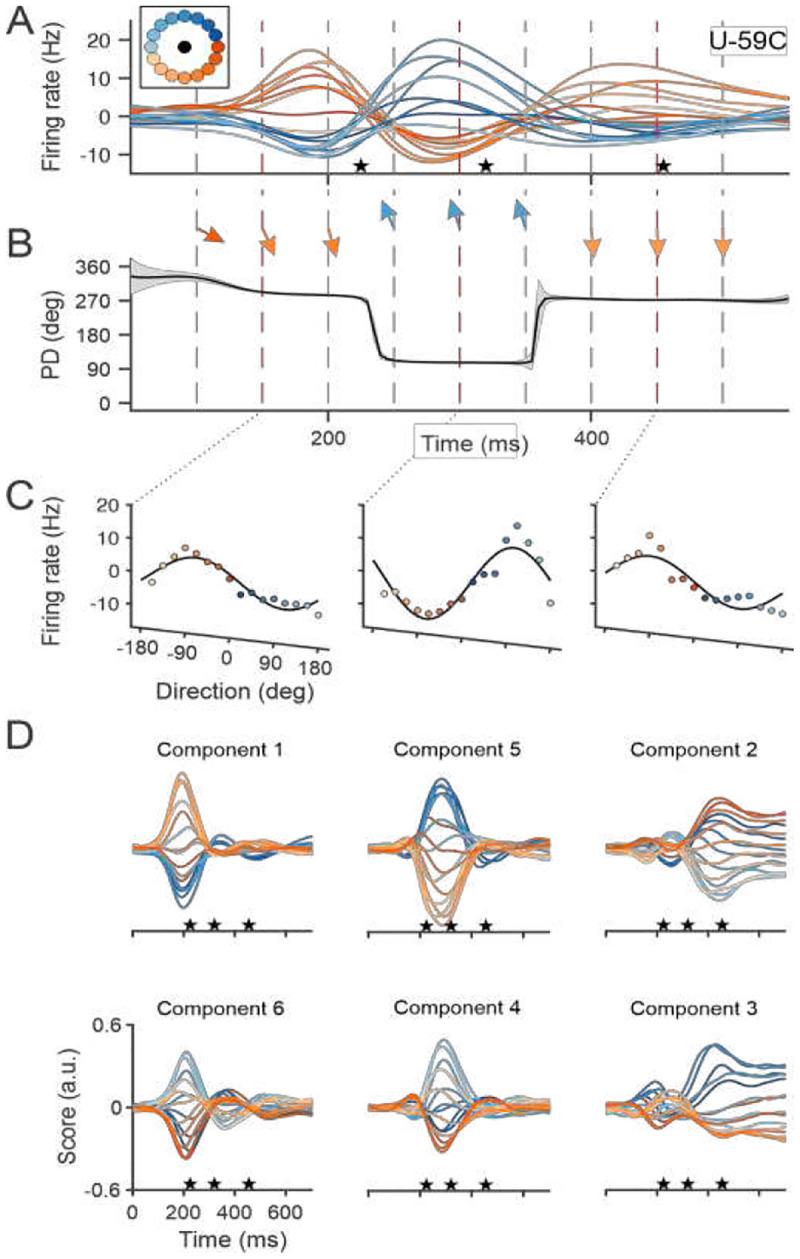
Multiple states in a single reach. (**A**) Trial-averaged firing rates from a single motor cortical unit, U-59C (Unit 59, Monkey C) during reaching in 16 different directions (color-coded inset). The mean firing rate across targets at each time point (non-directional component) was removed. (**B**) Preferred directions changed episodically. Preferred directions are also symbolized by the arrows. (**C**) Tuning functions at different time points (150, 300, 450 ms) during the reach. Firing rates are cosine-fit to the data. (**D)** rPCA scores from Monkey C. The six highest scores are plotted after Promax rotation. Scores are color-coded by movement direction. The profiles in each column have peaks at about the same time in the trial. The time axis begins at movement onset. The stars show the average time of movement onset, peak velocity, and movement completion, respectively.

Directional tuning during reaching has been shown to be non-stationary^37–42^, as tuning functions can change in a subset of neurons during a reach, especially as the movement begins. Recent work^43,44^ shows that while these tuning functions may change episodically during the reach, they are stable within each episode. Motor cortical neurons typically have one, two, or three modulation episodes during a reach^38,39,43^. We use the firing rates of Unit 59 recorded from Monkey C (also shown in^43^) as an example of modulation described with a three-epoch template (Fig. 1A). The firing rates of this neuron, like other neurons with multiple modulation epochs during a reach, are described with a tuning function that changes rapidly between epochs (Fig. 1B). The amplitude of the modulation profile in each epoch is cosine-tuned (Fig. 1C). These modulations are well fit with Gaussian profiles and, across neurons, their peaks are confined to one of three epochs during the reach^43^. This temporal consistency suggests that these modulations are common to the population *en masse*. The population-wide consistency of neuronal modulation and the associated changes in tuning suggest that epoch-specific signaling acts as a common factor across neurons responsible for these changes. Separate population modes in each epoch can be identified using analytical methods to extract individual patterns of correlation between the firing of neurons recorded together.

### Correlational structure between neurons reveals successive states

Principal component analysis (PCA) is commonly used for describing patterns of neuron-neuron correlation within a population of neurons from unitary recordings made with microelectrode arrays^45^. Standard PCA optimizes components that maximize the variance accounted for across the entire data set, which can reduce the explanatory value of the analysis. Instead, we are interested in finding a sparse set of components, defined by particular combinations of neurons, and applied a rotation to standard PCA^46^ (rPCA – Methods, Fig. S3) to find discrete patterns of population correlation^47^. The projection of the data points onto each axis is the *score* of the component and can evolve through time (Fig. S3B, C).

We used rPCA to analyze the populations of cortical activity we recorded from two monkeys and found that six components accounted for 80% of the variance in population firing rate for Monkey C (86% for Monkey N). The rPCA scores from Monkey C are shown in Fig. 1D. The single-peak amplitude of each component across movement directions is cosine-tuned. Pairs of scores peaking at the same time are plotted in the columns. The timing and shape of the profiles tend to match those of the modulated firing rates of single neurons^43^ and align with a set of behavioral events: movement onset, peak velocity, and movement offset (defined here as the beginning of the target acquisition process). The same results were found for data collected from Monkey N (Fig. S4). The link between the pairs of scores and their corresponding behavioral event was strong. rPCA components were calculated for individual trials and the timing of the component peaks was well correlated with that of the corresponding behavioral event (Methods: rPCA components vs event times and Fig. S5).

The term “state,” when applied to a system, often refers to the values taken on by the components being considered. A slightly different meaning is often used in network science^48–53^, where “state” refers to a period of stability signified by a consistent pattern of correlation between components. It is these network states that are found with rPCA. This result conforms to network dynamics composed of a series of state transitions aligned with the behavioral events taking place in a single reach.

**Figure 2.**
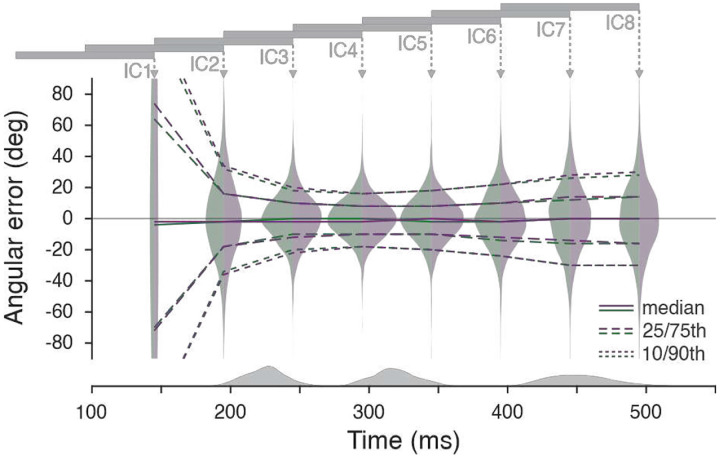
Single-trial reaching decoded with firing rates inferred by LFADS - a dynamical systems model. The angular error between true and decoded movement directions (Monkey C) is shown for eight LFADS models that differed only in the 100-ms segment (gray horizontal bars) of recorded population activity used to set each model’s initial conditions (ICs). These IC windows were spaced 50 ms apart, and each violin plot is positioned at the end of its corresponding window. Green violins report angular error for decoded velocities until peak velocity, and purple violins report error after peak velocity (see Methods). Solid lines indicate the median angular error across trials; dashed and dotted lines indicate the 25th/75th and 10th/90th percentiles, respectively. Gray histograms at the bottom show the trial-by-trial distributions of movement onset, peak velocity, and movement offset. Target onset occurred at 0 ms for all trials. Models whose initial conditions were inferred from neural activity preceding movement onset produced substantially larger decoding errors (e.g., second violin from left) compared with models initialized using neural activity after movement began (e.g., fourth violin; Wilcoxon signed-rank test on absolute angular error, p < 0.001). A similar finding was evident for Monkey N (Fig. S6).

The basic assumption of the current dynamic system approach^11^ is that a pre-movement population state acts as the initial condition for the autonomous generation of the ensuing movement. We explored this assumption by choosing different reach periods of neural activity as the initial condition with which to build dynamic models of the entire reach. If the basic assumption is valid, initial conditions from pre-movement data should yield predictions that are as accurate as models build from later portions of the data. Eight LFADS (latent factor analysis via dynamical systems) models were trained to generate firing rates during the task using data from eight different periods for the initial conditions (Methods). Results from Monkey C are shown in Fig. 2. When firing rates during the reaction time were used to set the initial condition (IC2, second violin from the left), the decoding error was large compared to intervals chosen during the movement (IC3–6); this finding indicates that the pre-movement data leads to a poorer dynamical model than firing rates during the movement.

### Hybrid Network Models

Our findings, to this point, show that during a reach, motor cortical activity takes place as three separate processes linked to behavioral events instead of the single autonomous process proposed by advocates of the dynamical systems perspective. Furthermore, this perspective dictates a role for pre-movement activity in initializing the dynamics of the system, which was not supported by results from the LFADS models. A third condition of this perspective is that the activity during the reach is autonomous (that is, it takes place only within the motor cortex via neuron-neuron interaction). The general model for a dynamical system (Eq. 1) encompasses two forms of input, intrinsic, r(t) and extrinsic, u(t). The autonomous aspect of the dynamical systems perspective considers only intrinsic input in the absence of extrinsic contributions (u(t)=0).


Eq. 1 $$\dot{r}(t)=f(r(t),u(t))$$

Where:

r is a vector composed of neuronal firing rates;

$\dot{r}$ is the change in firing rates over time;

u is the external input;

f describes how intrinsic and external input contribute to the change of firing rate.

To describe the effect of extrinsic input on neural dynamics during reaching, we built two HNNs that were driven by continuous extrinsic input, one with recurrent connectivity and the other with simple feedforward architecture, to probe plausible mechanisms for the dynamics of our data. The latter model was a spiking neural network. Both were trained to reproduce the firing rates of the individual cortical neurons recorded in our center-out experiments. We assumed that the cortical population we sampled was driven by common input with Gaussian profiles resembling those of the rPCA scores (e.g., Fig. 1D). Input neurons were divided into three groups, one for each episode of modulation, with their peaks aligned to the start of movement, peak speed, and movement offset in individual trials. Each group consisted of directionally tuned neurons with preferred directions evenly distributed around a circle. This type of input is plausible since many projections to the motor cortex are cosine-tuned to movement direction^54^.

**Figure 3.**
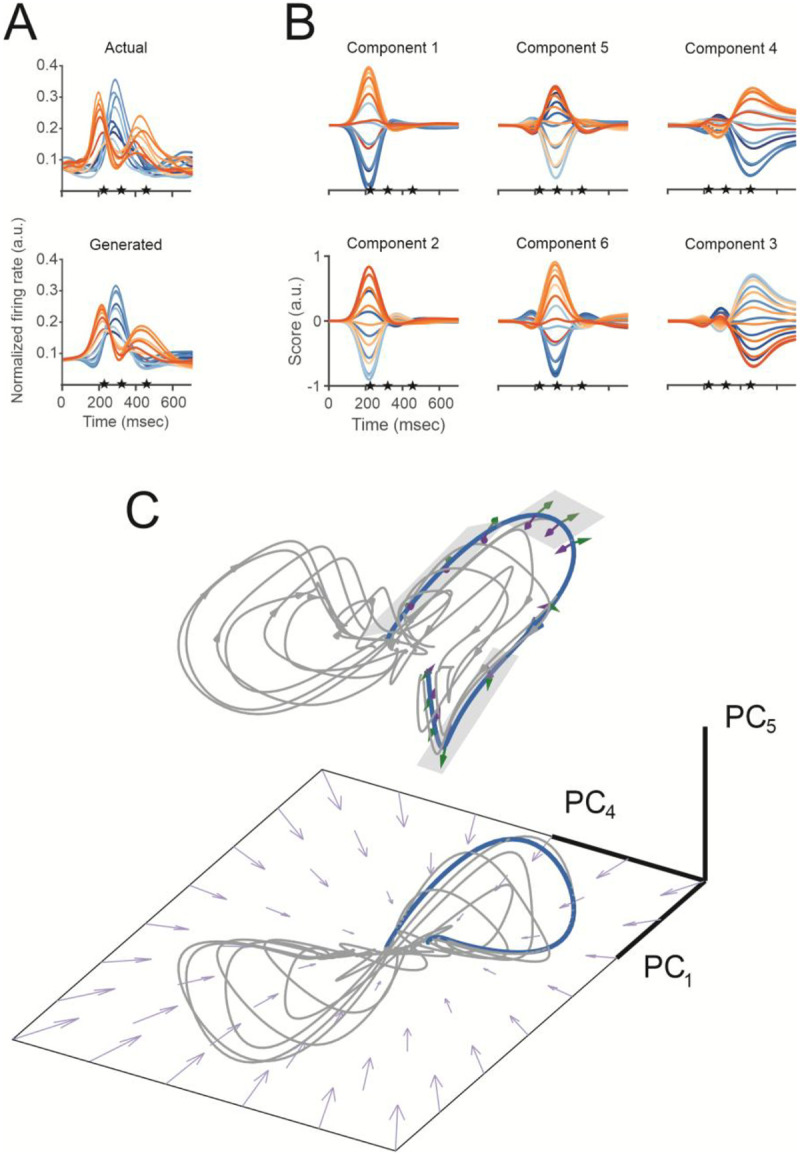
Recurrent neural network. (**A**) Trial-averaged firing rates of Unit 59, Monkey C. Actual (top, U-59C) and model-generated (bottom, RNN-59C) firing rates. Traces begin at target onset. Stars correspond to movement onset, peak speed, and the end of movement. (**B**) Six highest rPCA scores of RNN units after training. (**C**) Neural dynamics of RNN in a 3-D subspace (RNN State Space Trajectory-Suppl. Material). A neural trajectory, composed of rPCA scores 1, 4, and 6 from **C**, is plotted for each of the 16 reach directions trials (target onset to target acquisition). Trajectory components are illustrated with purple (recurrent activation) and green (activation from extrinsic neurons) arrows. Arrows on the plane indicate the change in network activity driven by intrinsic activation at each point on the manifold.

We were able to separate the effect of intrinsic and extrinsic input on our observed neural activity using a recurrent HNN (Fig. 3) and found that the extrinsic input was primarily responsible for imparting the modulated firing in our data. To further substantiate this finding, we applied the modeled extrinsic input to a spiking HNN with only feedforward connections to neurons trained to have the same firing rates as those in our data. This network, without an intermediate layer, also replicated our data. Analysis of this network showed that a combination of specific input neurons contributed to each state.

### Recurrent HNN

We consider the neural activity recorded from primary and premotor cortices to be a sample from a large, interconnected network of neurons involved in generating behavioral output. With this consideration, we modeled the activity during our experiment by embedding this sample as a subset of neurons in the hidden recurrent layer of an artificial neural network. The recurrent layer acts as a local network containing the neurons whose activity we recorded empirically. All neurons in this layer receive input both from intrinsic neurons and from those that are extrinsic, as described in the previous paragraph. This network model allows us to distinguish the effect of each type of input on the empirically recorded activity.

#### Extrinsic and Intrinsic Input

Recurrent neural networks can be trained to produce temporal patterns of output using only internal feedback pathways of different duration. Such a model was developed by Sussillo et al.^55^, to show how recurrent neurons, provided with *transient directional input* to set an initial condition, could produce subsequent temporal patterns of muscle activity during reaching. We used an RNN with a similar architecture to instead show how *ongoing extrinsic input* can interact with recurrent activity to generate models of the firing rates we observe in our data (Methods). After training, we found that the firing rates of neurons in the recurrent layer were cosine-tuned, that the models of the empirical firing rates were highly accurate (mean correlation = 0.91, Monkey C, 0.96 for Monkey N; Fig. 3A, S7A), and that the overall population underwent the same state transitions as those in our data (Fig. 3B).

The interplay between intrinsic and extrinsic factors can be described using the correlational structure of the RNN activations, x(t) (Eq. 2), as they evolve during the movement^56^. To visualize the dynamics of these correlation patterns, we carried out rPCA on the activations and plotted their projections on three of the axes to form neural trajectories (Figs. 3C, S7C, Methods). Since the profiles of these scores overlap in time, the resulting neural trajectory, when plotted in a 3D manifold, is curved. $\dot{x}(t)$ is formulated as a linear combination of input from recurrent connections and external inputs, so that the tangent along the trajectory can be divided into components that are intrinsic (purple arrows) and extrinsic (green arrows). While the intrinsic dynamics due to recurrent connections tend to pull neural trajectories toward the center (a point attractor), the external inputs drive individual neural trajectories in different directions (green arrows) during individual epochs of the movement. Each trajectory segment in these different directions corresponds to one of the network states (gray planes in Fig. 3C) and shows that the extrinsic input is driving the changes in dynamics of the model. The same results were found for data collected from Monkey N (Fig. S7).

#### Input Contributions

**Figure 4.**
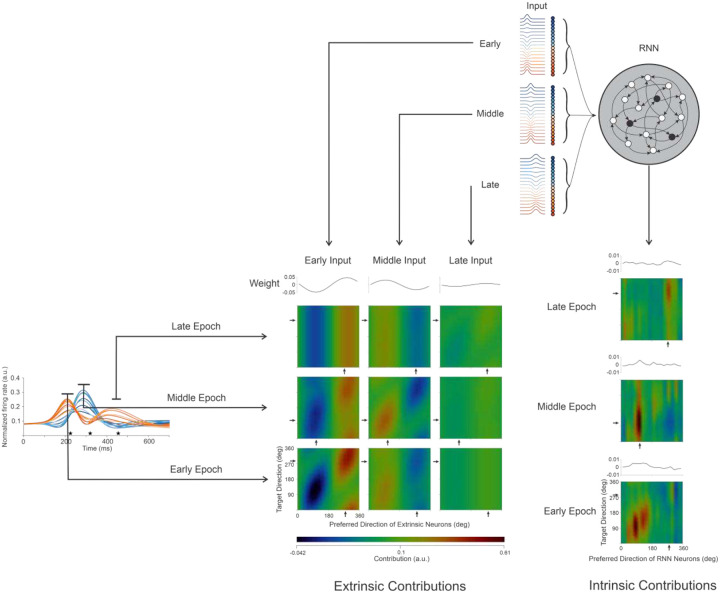
Epoch- and movement-specific contributions to the firing of Unit RNN-59C. The RNN cartoon shows how the empirical neurons (black) were included in a hidden layer with artificial neurons (white). Three groups of external input neurons, with firing rates modulated in a single epoch were fully connected to the neurons in the hidden layer (color-coded by preferred direction). Heatmaps are composed of the contributions from input groups (horizontal axis) in each direction of movement (vertical axis). A heatmap row corresponds to an epoch. The traces above each column show the direction-specific weights for that input group. Pixels of the heatmap show the total contribution from the units in a subgroup containing input neurons with similar tuning during an epoch of all trials with the same movement direction. The arrows on the vertical and horizontal axes indicate the preferred direction of RNN-59C for that epoch. The left table of heatmaps shows the contributions from the extrinsic neurons to the network. In the panels where the input was modulated in the corresponding epoch (early-early, middle-middle, and late-late), there is a prominent diagonal banding, showing that these contributions could impart directionality to the firing rate of the output neuron. To calculate the contributions for the intrinsic input, the RNN units are divided into18 groups, according to their preferred directions in each epoch. The vertical stripes in each of the intrinsic heat maps (rightmost column) indicate that they are not specific to movement direction.

The defined connectivity of the trained RNN, combined with the prominent tuning of the neurons in the network, make it possible to investigate how inputs are combined to produce the firing-rate patterns of the individual neurons in our data set. To understand how directionality is imparted to the output neurons serving as models of our empirical results, we analyzed the differences between the effect of contributions that are intrinsic (recurrent layer) and extrinsic (input groups) (Eqs. 4, 5) to the modeled firing rates. The contributions to the firing of RNN-59C are visualized in separate heatmaps (Fig. 4) for the intrinsic and external inputs in each epoch (see Methods and Fig. S8 for another example unit). Since both input sets had cosine-tuned firing rates (prescribed for the extrinsic units, calculated for those in the recurrent layer), they were grouped by preferred direction (heatmap columns) and evaluated in each movement direction (heatmap rows).

The preferred direction of the input in each column coincides with the direction of movement along the diagonals of the heatmap. As expected, this is where we see the largest positive and most negative contributions for movement toward the preferred and anti-preferred directions, respectively. This expectation holds for the extrinsic inputs, but not for those that are intrinsic. In the early epoch (Fig. 4), the positive and negative intrinsic contributions are reversed, which, by itself, would lead to tuning opposite to the output unit’s preferred direction in that epoch (compare intrinsic heatmap, Fig. 4 bottom panel, rightmost column to corresponding extrinsic heatmap bottom row, left column). The intrinsic heatmaps for the other epochs have vertical bands which are positive for input that matches the output preferred direction and negative bands in the anti-preferred direction. Since there is a similar positive and negative contribution of input for each movement direction, these would tend to cancel out, again showing that the intrinsic input cannot account for output directionality.

The strong contributions along the diagonal promote directional tuning in the output unit. These results and those of two additional analyses, Input Correlation (Fig. S9) and Input Dropout (Fig. S10), show that the extrinsic input was the dominant factor governing the directional tuning in the RNN model across all units in our data.

### Spiking neural network

Motor cortical activity is a function of both intrinsic and extrinsic connectivity. Nonetheless, based on the results from the RNN, we asked whether *extrinsic* input, by itself, could produce the temporal patterns of firing we observed in our data. Again, we assumed that the three profiles uncovered by rPCA (Fig. 1D) signified common input and modeled this as three groups of 90 tuned neurons modulated at different points during the reach. Each neuron’s firing was driven by a Gaussian temporal profile. In addition to these three sets of directional input, a fourth group was used to impart a non-directional speed offset^23,57^ to the firing rates (Fig. S11A). This group was driven by the movement speed profile shifted 50 ms forward with gains that varied from −0.5 to 1, across the 90 neurons.

**Figure 5.**
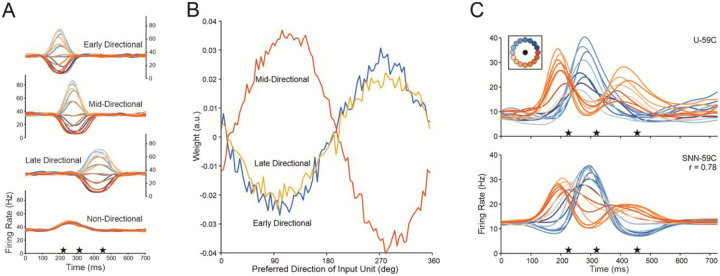
Spiking neural network. (**A**) Trial-averaged firing rates for one example input neuron in each group. The three example neurons were tuned to 180°. Stars indicate average time for movement onset, peak speed, and movement completion. The non-directional example neuron had an amplitude coefficient of 0.25. (**B**) Weights for the directional input units to modeled unit (SNN-U59C). (**C**) Actual and predicted modeled firing rates for Unit 59. The stars mark the behavioral events as in **A**.

For an individual trial, the drive to each input was transformed to spike trains (visualized as firing rates in Fig. 5A) using the integrate-and-fire algorithm in the Brian2 simulator^58^. On individual trials (repetitions 1–20) during training, we applied Hebbian learning using the spike occurrence times of the recorded units and those of all input units to find input-output weights (Figs. 5B, S11B). After training, modeled firing rates of the output neurons were generated with the SNN by aligning the input groups to the behavioral landmarks during the trials (21–40) not used for training (Figs. 5C, S12). Firing rates of the recorded neurons were well fit by the modeled output (r=0.71 across all units for Monkey C and r=0.69 for Monkey N). However, two factors led to a slight reduction in SNN performance compared to the RNN: the SNN struggled to capture shifts in baseline firing rates and it had limiting fidelity when the recorded unit’s modulation did not align with the time course of input unit modulation. Population dynamics of the modeled data matched that of the actual data (compare Fig. 7A to Fig. 1D), showing that the model captured the network state transitions. The common input, by itself, drives both the predicted firing rates and the state transitions of the network.

#### Statistical structure of synaptic integration

**Figure 6.**
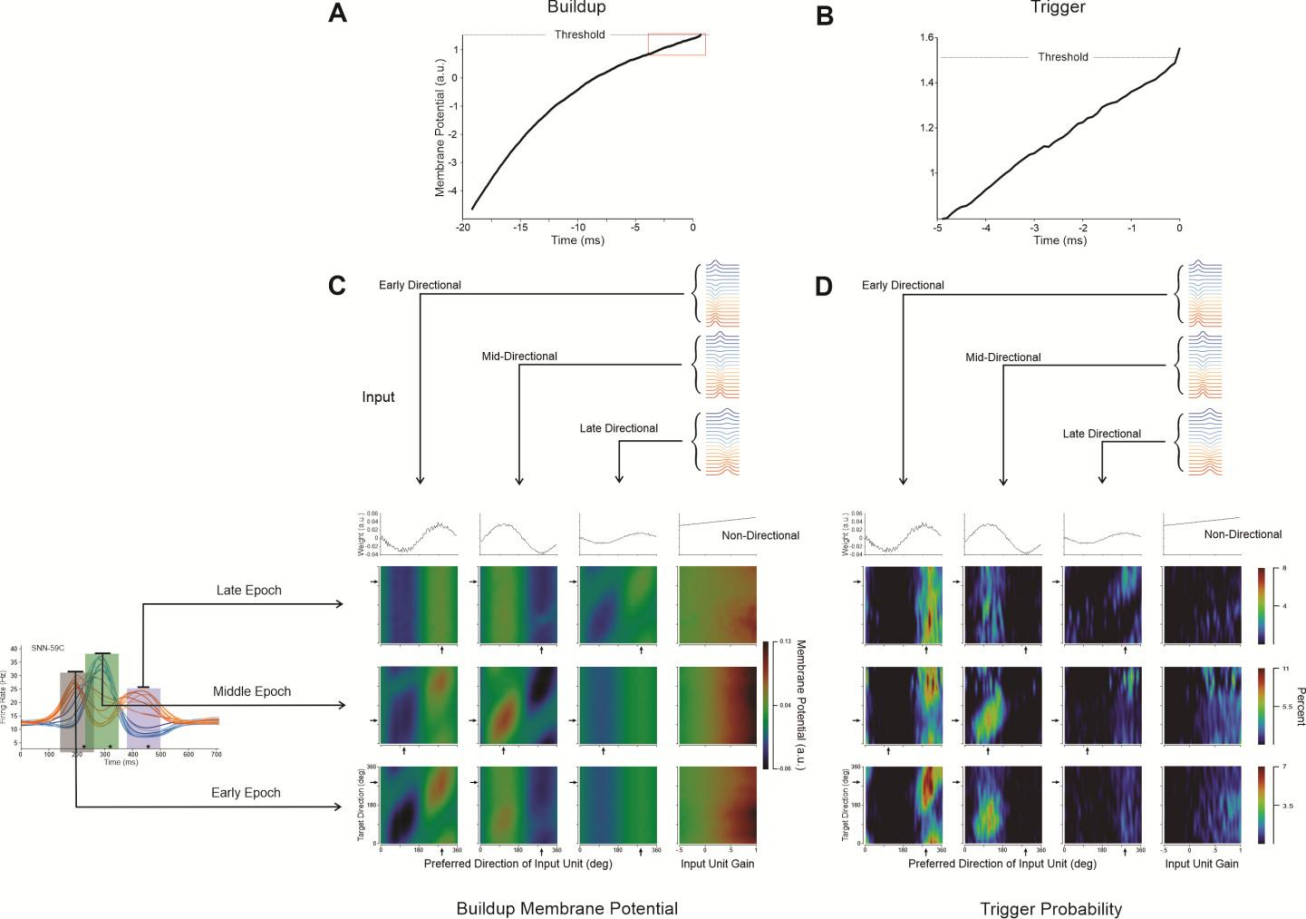
Input contributions to output spiking. Exemplified with SNN-59C (**A**) Spike-triggered average of membrane potential. The membrane potential of the modeled unit in the 20 ms buildup period before an action potential was averaged across its spikes in the middle epoch time zone (green rectangle in firing-rate histogram) for a reach at 112 degrees. (**B**) Spike-triggered average of the 5 ms before the spike ending with the trigger event. (**C**) Heatmaps of input-driven change in membrane potential during the build-up before an action potential. Rows come from the firing during the early, middle, or late epochs during a reach. Columns correspond to input from one of four groups— early, middle, or late directionally modulated and non-directional (speed modulated). The weights for each input group are shown in the top row. (**D**) Probability of an input neuron acting as a trigger (input spike 0.1 ms before the output spike). Columns correspond to the input groups, and the other rows correspond to the epochs shown in **B**. Arrows indicate the epoch-specific preferred direction of the modeled unit (SNN-59C).

The SNN model explicitly captures the postsynaptic effects from presynaptic connections. Using a single example neuron (SNN-59C), we used spike-triggered averaging^59^ to show a gradual rise in membrane potential 20 ms before an output spike (Fig. 6A), followed by a sharp increase just before spiking (Fig. 6B). In one of the few behaving primate studies in which intracellular recordings were made in the motor cortex^60^, it was found that the contribution of input-driven postsynaptic potentials to the “prethreshold ramp potential” could be distinguished from the triggering of the action potential. Accordingly, we considered synaptic integration in two intervals before an output spike: *buildup* – 20 ms (Figs. 6C) and *trigger* – 0.1 ms (Fig. 6D). These analyses were carried out separately in the three behavioral epochs of modulation.

The correlation between firing rates from a pair of neurons may be due to two factors: their covariation of firing rates and the synaptic connectivity between them. Covarying firing rates can induce correlation in the absence of physical connectivity. With empirical recordings, this distinction is difficult to recognize, as “functional connectivity” can vary during behavior when separate input groups become differentially active in different contexts^61^. However, in network models such as the SNN we are using, the physical connectivity is explicit and the consequence of an input can be measured directly by the change in membrane potential it causes. The potential change per input spike is proportional to its input-output weight. Input neurons fired most rapidly when their preferred directions aligned with the reach direction, forming a diagonal pattern of membrane potential during the buildup for those heatmaps showing the input groups that matched the behavioral epoch (Fig. 6C). This SNN pattern of input contributions to membrane potential closely match the extrinsic activations in the RNN (Fig. 4), suggesting consistent input-output patterns across architectures. The diagonal pattern of contributions in the SNN heatmap was evident across the activity in the data sets from both monkeys (Diagonal Test – Methods).

The probability of an input triggering an action potential is shown for the same output unit in Fig. 6D. These probabilities were more punctate than the contributions to membrane potential during buildup. The input weight was a predominant determinant of trigger probability. For instance, neurons in the early input group tuned to 280 degrees had large weights and were most likely to trigger an output spike for each epoch, even when those units were only firing at their background rates during the late epoch.

Although neurons in the late input group fire much faster in the late epoch, their relatively small weights (weak connectivity) make them unlikely to trigger an output spike. This finding was consistent for our entire data set (Fig. S13).

During buildup, non-directional inputs, aligned with reach speed profiles, contributed primarily during the early and middle epochs. These inputs had a large effect on neurons with single-epoch firing rate modulation (Unit SNN-48N in Figs. S12 and S14). Whereas these inputs tend to modulate the firing rate of neurons in the middle of the reach, the directional tuning of each output unit comes from the three sequential sets of directionally tuned input neurons. The non-directional inputs were unlikely to trigger an action potential in the output neuron (right column of Fig. 6D).

In summary, the SNN modeling shows that directionally tuned input neurons with preferred directions near the movement direction contribute to the buildup of an output neuron’s membrane potential toward threshold, while those with the largest weights (tuning matching that of the output neuron) are most likely to trigger the spike.

## Discussion

Set-and-forget schemes have been a significant component of the motor control literature for more than half a century. A current version, the dynamical systems perspective (reviewed in^62^), is predicated on three basic suppositions: that a single autonomous control process is released at movement onset; that pre-movement neural activity sets an initial state for the control process; and that extrinsic input is not a causal component of ongoing neural activity. Here we counter each supposition with conflicting findings:

### Reaching is not a single process.

**Figure 7.**
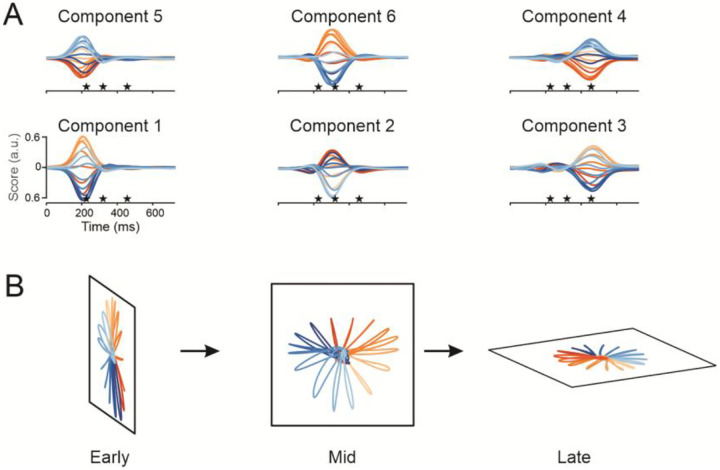
Discrete dynamic components (data from the SNN-predicted firing rates--Monkey C). (**A**) Three pairs of rPCA scores defined by coincidence of their peak values. The stars denote the mean behavioral landmarks of movement onset, peak speed, and the end of movement. The traces begin when the target was presented. (**B**) Each of the three pairs was plotted on separate axes of a plane to form a manifold, showing how the correlated structure in an epoch varies during movements in different directions. The orientation of the three planes was chosen to emphasize the state transitions associated with each epoch. Movement direction is color-coded.

Proponents of the dynamical systems perspective point to what they term “rotational dynamics” in their finding that PCA components form curved neural trajectories through state space. These trajectories were interpreted as a signature of a single, first-order oscillatory dynamical process^63^. However, PCA applied to neuroscience data often leads to “phantom oscillations” caused by temporal shifts of components^64^. Our study demonstrates that these curved trajectories are formed from pairs of overlapping temporal components. We used rPCA, in which a rotation of the standard PCA produced sparsely loaded eigenvectors and succinctly interpretable components. With this analysis, we identified distinct pairs of contemporaneous latent variables that appear at distinct behavioral events. Coincident component pairs calculated from the SNN simulations closely matched those of the empirical data (Figs. 1D, 7A). When the sequential pairs of components are plotted on a sequence of manifolds, they form straight center-out neural trajectories (Fig. 7B), consistent with a series of separate cortical states during a reach. Our results with this methodology show that, instead of a single process, cortical activity evolves through separate discrete network states associated with different components of the reach.

The finding of distinct behavior-related neural states has direct bearing on previous experiments that explored cortical dynamics using correlation patterns in recorded neural populations. For instance, in a recent study^65^, monkeys used decoded cortical activity to perform a center-out reaching task; neural trajectories were then presented to the monkeys, who needed to follow the trajectory by modulating their cortical firing. The monkeys were successful when starting from the beginning and proceeding forward through the trial but were unable to begin the trial at the end of the trajectory and move to its origin. The investigators concluded that this inability was due to an intrinsic dynamical structure that is “highly conserved and not readily changeable,” with the implicit assumption that the neural trajectory was derived from a single dynamic process. We propose another explanation: rather than acting as the source of movement dynamics, the isolated activity observed in the motor cortex is only one part of a large system that reflects the ongoing action in general, which is behavior driven. In this context, our findings of separate behavior-linked processes suggest that the task failure is due to the inability to use a control process for one behavior to supplant a process for a different behavior. The process at the beginning of the movement, when the arm has yet to move and the eyes saccade to the static target, is completely different from that which occurs at the termination of the reach, when small sub movements of the hand are taking place with foveated visual feedback. In short, reach termination cannot precede reach initiation.

### Reaches are not fully determined by a pre-movement initial state.

We assessed the precedence of pre-movement activity in setting the initial condition for subsequent deterministic dynamic unfolding of cortical activity during reaching. Using a latent factors model seeded by an initial condition^66^, we found that pre-movement activity used to set the initial condition gave a poorer prediction of the upcoming reach than activity selected from later portions of the reach. While motor control requires prediction, the premise of entirely predetermined movement conflicts with behavioral results showing that reaching is composed of distinct behavioral components^12,67^ and with the need for the system to quickly compensate for external perturbations and inherent noise^7,18,68–72^. A more comprehensive view of reaching encompasses the mechanical state of the body. From this view, the dynamics of the middle epoch begins as the arm decelerates after its initial acceleration followed by the dynamical sequence to acquire the target, which begins as the arm slows near the target. Reaching takes place as a chain of dynamic processes, with each link triggered by the previous process.

### Extrinsic input is vital.

We built realistic neural networks models to explore the interaction between intrinsic neurons and extrinsic inputs. First, we used an RNN to examine the relative effects of intrinsic and extrinsic input on the firing rates of the neurons we recorded empirically. This model showed that the extrinsic input was responsible for generating the directionality of the recorded neurons. Based on this result, we asked whether a simple feedforward spiking neural network (SNN) driven *only* by extrinsic input could account for the empirical activity in our recorded data. The SNN accurately modeled the firing rates of the recorded neurons as well as the individual temporal patterns of correlation in the overall population, supporting the role of continuous input to motor cortical areas during reaching.

Our results emphasize the importance of including ongoing extrinsic input in the consideration of cortical dynamics. We show that extrinsic drive, by itself, can produce the firing rates we observed in much of our data. Input was organized into separate groups of temporal modulation, which was integrated into output patterns of modulation. State transitions were driven by successive groups of input neurons. Input neurons with similar tuning fire rapidly for movements in their common preferred direction. However, each input neuron’s effectiveness varies with its input-output weight. We divided synaptic integration into two components: buildup and triggering. Contributions to membrane potential during buildup were governed both by the firing rates and the weights of the input neurons, while the neurons that triggered spikes were those with strong connections to the output neuron regardless of firing rate. Input neurons contributing to the membrane buildup had a broad range of preferred directions, while those that triggered the spike were tuned to a narrow range around the output neuron’s preferred direction.

The distinction between buildup and triggering is consistent with results from an *in vivo* mouse study in which the membrane potentials of visual cortical neurons were recorded as oriented visual input was presented^73^. A wide range of orientations increased the membrane potential, but only those that closely matched the output orientation triggered a spike. The buildup and trigger phases of spike generation have been considered in a computational model showing that combined excitatory and inhibitory input contributions (during buildup) can set the “working point”^74^ of an output neuron to a critical range, making it sensitive to additional input (triggers) that can change its effective connectivity^61,75^. The importance of the buildup trajectory was emphasized in a model describing how widespread polysynaptic signaling can generate pairwise correlation of firing in the motor cortex^76^. In this study, integration of synaptic input changed a target neuron’s “gain,” a metric of its pre-threshold excitability. In turn, the effectiveness of a particular input neuron in changing the firing probability of an output neuron is a product of its synaptic weight and the current gain of the output neuron. Variations in the composition of the contributions during buildup can explain why ineffective inputs during one state can become effective in another.

Beyond a comparison of input sources, we were able to describe aspects of the network mechanics that govern the generation of the network activity recorded in our experiments. We found that correlations between the firing of neurons, detected through dimensionality reduction, arise largely from extrinsic input. Anatomical projections to an output neuron may be hardwired, but they will not be connected functionally unless they are active^61^. Dimensionality reduction analyses, such as PCA, are based on neuron-neuron correlation. While firing-rate correlation between a pair of neurons might imply that they are directly connected^55,77^, this is unlikely to be a factor in the recorded data, given the spacing between electrodes of the Utah probe^78,79^. Multiple studies have shown that polysynaptic and shared input can drive these patterns^54,75,76,80–84^. In our models, these correlations reflect directional tuning as a global property of network function^53,78,85,86^. Our analytical results suggest that episodic states correspond to behavioral events: movement onset, peak speed, and target acquisition^12,87,88^. In particular, late epoch, target-related activity is diminished when visual accuracy requirements are removed^43,89^. It is generally agreed that state transitions in biological neural networks are synaptic phenomena^52,90–94^, but the organization of events leading to a state transition remains unclear. We are just beginning to consider the structure of these synaptic mechanisms in order to explain how the same population of neurons can participate in multiple neural operations—a primary characteristic of distributed systems^95^.

It is important to add intrinsic connectivity to the SNN to investigate the interplay between elements within a population of neurons and sources of external drive in a more complete dynamical systems model (e.g., Eq. 1). Multiple models^36,55,96^ and empirical recordings of localized cortical activity^90,91,97,98^ have shown how neuron-neuron interaction contributes to network functionality. A recent study using optogenetic manipulation of extrinsic projections to the motor cortex^99^ not only showed the importance of extrinsic input during reaching, but was able to differentiate the effect of this input on localized intrinsic activity.

Recognizing patterns of neuronal activity within a neural population is a first step in characterizing the complexity of network functionality^61,100^. Future studies using hybrid neural networks are likely to push the field forward. The realism of these models could be enhanced by adding biological properties of synapses such as channel conductance, transmitter species, receptor properties, and structural anatomy. Patterns of complex input can supersede the simple single-epoch modulation patterns in our current model. The complex modulation of input firing rates in the presence of modulatory input and/or rapid molecular modification of synaptic transmission^101^ are aspects of realism that likely will be important features of future modeling efforts.

As system models become realistic, the concepts of input, output, and modularity become more obscure^102^. By redefining network boundaries (for instance, by expanding the model well beyond the motor cortical areas), the designation of intrinsic connectivity and extrinsic input will be recognized as abstract constructs. These new models will expose the crucial, widespread interdependencies between the nervous system and the innumerable somatic, social, and physical aspects of the world around us. With all-encompassing artificial neural networks on the horizon, we have the prospect of building models that can account for these myriad factors, which in turn, will help resolve a variety of neuroscientific controversies as well as move the field toward more realistic concepts of system functionality.

## Methods

### Behavioral task

Two male rhesus macaques (monkeys C and N) performed a center-out reaching task using a virtual-reality setup. Each monkey sat in a primate chair with one arm restrained. An infrared marker was placed on the wrist of the freely moving arm. The position of the marker was sampled at 60 Hz (Monkey C) or 100 Hz (Monkey N) (Optotrak, Northern Digital) and projected as a spherical cursor on a 3D monitor (Dimension Technologies). At the start of each trial, a spherical target appeared in the center of the virtual space and the monkey was required to hold the cursor within the target for 200–300 ms. The center target was then extinguished and a peripheral reach target (radius of 10 mm for Monkey C, and 6 mm for Monkey N) was presented at a position randomly selected from 16 predefined locations. The 16 peripheral targets were radially arranged around the center target on a 2D virtual plane in front of the monkey (target-to-center distance of 7.4 cm for Monkey C, and 6.5 cm for Monkey N). Trajectories and speed profiles of the reaching movements are shown in Supplementary Fig. S2. A liquid reward was administered after the monkey reached and held within the 3D target sphere for 200–400 ms. All procedures were conducted in accordance with the guidelines of the US National Institutes of Health and were approved by the Institutional Animal Care and Use Committee of the University of Pittsburgh.

### Neural recording

Monkey C was implanted with one multi-electrode array (96 channels, Blackrock Microsystems) in the arm area of the primary motor cortex contralateral to the freely moving arm. Monkey N was implanted with two arrays in the dorsal premotor cortex (Fig. S1). Single-neuron responses were isolated using the Offline Sorter program (Plexon Inc.). Ninety-three single units were recorded from Monkey C, and 113 units were recorded from Monkey N. The Monkey C data have been used in previous studies^43,57^; the Monkey N data are novel.

### Data pre-processing

To perform trial-averaging, center-out reaching trials were aligned at a series of behavioral landmarks, including peripheral target presentation, movement onset, maximum speed, movement offset, and target hold off. Movement onset and offset were defined as the times when speed reached 20% of the maximum. Trial alignment was implemented by fixing the number of time bins in the epoch between a pair of adjacent landmarks for all trials. The number of time bins in an epoch was determined by the average duration of that epoch across all trials divided by bin size (e.g., 5 ms per bin). The number of fractional interspike intervals in each bin was divided by the bin size to get firing rate^103,104^. The spike rates were smoothed with a Gaussian kernel (25 ms SD). For each neuron, firing rates were then averaged across trials with the same target (47 repetitions of each target for Monkey C, and 49 for Monkey N). The mean firing rate, over time and across all task conditions, was calculated for each neuron. Neurons with low mean firing rates (< 1.5 spikes/s) were excluded from subsequent analyses, leaving 67 units for Monkey C and 78 for Monkey N. A soft normalization^63^ was used to normalize firing rates, so that all neurons had similar ranges after the process. Hand position was spline-interpolated and then resampled to match the number of bins in each epoch. Velocity was then recalculated using resampled positions. All data processing and subsequent data analyses were performed using customized Matlab (Mathworks Inc.) scripts, except that the spiking neural network modeling (see “Spiking neural network” section, below) was performed with Python codes.

### Principal component analysis with rotation

Each trial-averaged firing rate data set was combined into a matrix of size c×t×n, where c is the number of conditions, i.e., movement directions, t is the number of time bins, and n is the number of neurons. Non-directional components were first calculated for single neurons by averaging the c firing rate profiles across conditions. These non-directional components were then subtracted from the profiles of each neuron to yield the directional components.

The directional and non-directional modulation of the recorded units were analyzed following the general approach of Suway et al.^43^ – the directional component was fit to a cosine function for each time bin and each unit, which uncovers the amplitude and fit $\left({\text{R}}^{2}\right)$ for directional modulation. One through four Gaussians were fit to this directional modulation profile. The number of Gaussians was chosen to fit the data well, and to cover the areas of data that had good cosine fits – with a penalty to discourage using additional Gaussian without explaining significantly more variance. We tested for the presence of modulation in the non-directional component by determining if the profile changed at least 1.5 Hz over the course of the trial.

Principal component analysis (PCA) was used to reduce the dimensionality of data. The previous firing rate data matrix of size c×t×n (with non-directional components removed) was reorganized to be matrix X of size ct×n, by concatenating firing rate profiles of all conditions for individual neurons. Each row of X was a 1×n vector of firing rates from the neuronal ensemble, and there were ct samples from all time bins and conditions. PCA found a set of n orthogonal axes, i.e., principal components (PCs), in the n-dimensional firing rate space, such that the top k(k<n) PCs defined a subspace that explained the most variance of the data. The data matrix X was accordingly decomposed as:

$$X=SU^{T}$$

where matrix U of size n×n had n eigenvectors as columns, $U^{T}$ was the transpose of U, and matrix S of size ct×n contained PC scores. Each row of S was a 1×n vector of scores, which were projections of the corresponding row of X onto the n eigenvectors. Columns of U were ordered by the amount of data variance explained by PCs, from high to low. By keeping only the top k PCs, we reduced the dimensionality of X from ct×n to ct×k, and had

$$X_{red}=S_{red}U_{red}^{T}$$

where $X_{\text{red}}$ of size ct×n contained reconstructed firing rates using top PCs and scores, $U_{\text{red}}$ of size n×k had the first k columns of U, and $S_{\text{red}}$ of size ct×k had the first k columns of S. In our analyses, we chose k to be 6, thereby accounting for 80% of the variance of firing rates in the population for Monkey C (86% for Monkey N).

To reinforce simple structure and improve interpretability, Promax rotation was applied on the $S_{\text{red}}$ matrix of PC scores. A target matrix using the Equamax method was found first. A transformation matrix, R, of size k×k, was then calculated such that $S_{\text{red}}R$ conformed best to the target matrix with respect to the sum of squared errors criterion. $X_{\text{red}}$ can be decomposed as

$$X_{\text{red}}=\left(S_{\text{red}}R\right){\left(U_{\text{red}}R^{-T}\right)}^{T}$$

where $R^{-T}$ was the transpose of the inverse matrix of $R.U_{\text{red}}R^{-T}$ was a matrix of size n×k and its columns were the rotated eigenvectors (rPCs). $S_{\text{red}}R$ of size ct×k consisted of rotated scores (rPCA scores). The transformation matrix R was found using the Matlab rotatefactors function. Note that the rotated axes were not principal axes (or principal components) anymore, and they were no longer required to be orthogonal to each other. We refer to this PCA with rotation approach as rPCA.

We illustrate the difference between rPCA and standard PCA, using a toy dataset containing the firing rate profiles for reaching movements in 16 directions from three simulated neurons (Supplementary Fig. S3A). The firing rate of each neuron is directionally tuned during three temporal epochs centered at 0.3 s, 0.45 s, and 0.7 s, respectively. Around the center of each of the three epochs, a firing rate modulation “hump” (with the shape of the probability density function of a Gaussian distribution) was added to the constant baseline firing rate of each neuron. The amplitude of each hump equals the modulation depth (a scalar) multiplied by the cosine of the angle between the movement direction and the neuron’s preferred direction during that epoch. The modulation depth for each neuron in each epoch was randomly chosen from a uniform distribution between 0 and 1. The preferred directions of all neurons are either the same or 180 degrees apart during the same epoch, such that the neural trajectories during those periods of time follow straight lines (Supplementary Fig. S3D, seg. 2, 4 and 6). The preferred direction changed randomly between epochs. The width of the firing rate humps was determined by the standard deviation of the Gaussian distribution, which was chosen to be 0.04 s, 0.05 s and 0.09 s, respectively, for the three epochs. This created an overlap between two adjacent epochs, and therefore the neural trajectories tend to curve during these overlaps (Supplementary Fig. S3D, seg. 3 and 5).

The standard PCA could not isolate those directionally tuned modulation epochs from one another, since each PCA component was active in all three epochs (Supplementary Fig. S3B). In contrast, the rPCA successfully separated those epochs (Supplementary Fig. S3C) and the rPCA axes (black straight lines in Supplementary Fig. S3D) are well aligned with data samples from the non-overlapping portions of those epochs. Therefore, PCA with rotation was used as the default dimensionality reduction method in this study.

### rPCA components vs. behavioral event times

To determine whether the three rPCA-derived neural patterns (early, middle, late; columns of Figs. 1D, S4D) were expressed at consistent moments within individual reaches, we quantified when each pattern reached its peak activation on single trials. For each movement direction and each rPCA component pair, we first identified a “neural direction” in the corresponding 2-D rPCA plane (similar to those in Fig. 7B) by locating the point in the trial-averaged trajectory where that component pair reached its maximum amplitude. This point defined a vector $v_{i,k}$ (pattern i, target k) that was restricted to be between the peak modulations of the constituent components. We then projected the single-trial population activity onto the corresponding vector. These projections typically formed a single peaked time course, and the time of this peak provided an estimate of when the corresponding neural pattern was maximally active in that trial.

Because single-trial projections are usually noisy and sometimes lack a clear peak, we excluded targets whose peak amplitudes were too small or whose average peak timing deviated substantially from the expected timing based on the trial-averaged rPCA scores (Fig. S5A). For the remaining targets, we further removed outlier trials by retaining only those with peak times lying within the main cluster of the distribution (defined by the median ± a scaled interquartile range; Fig. S5B). After these exclusions, we compared the single-trial peak times with the timing of key behavioral events. Peaks from projections onto the early, middle, and late rPCA patterns were correlated with movement onset, peak velocity, and movement offset, respectively, allowing us to assess whether the temporal alignment observed in the averaged data was preserved at the level of single trials (Fig. S5C).

### Decoding movement velocity from the LFADS model

The latent factor analysis via dynamical systems (LFADS) model was used to infer single-trial neural population firing rates from recorded neuronal spiking activity^105^. LFADS starts with inferring the initial state for a recurrent neural network inside the model, and then the network operates autonomously (when no inferred input is included) to generate latent factor dynamics and, subsequently, neural population firing rates. For Monkey C and Monkey N, LFADS models were trained to generate firing rates during a 490 ms (and a 705 ms) period of each reach, starting from 100 ms after target onset. Eight LFADS models were trained, each using neural activity from a different period of the reach to set the initial state. These initial condition (IC) windows were 50 to 150 ms, 100 to 200 ms, 150 to 250, 200 to 300 ms, 250 to 350 ms, 300 to 400 ms, 350 to 450 ms, and 400 to 500 ms after target onset. [The LFADS model has 40 latent factors, and both the Encoder and Generator of the model have 100 recurrent units.] We then evaluated movement related information presented in these IC windows by decoding movement velocity using neural population firing rates inferred by corresponding LFADS models.

The first 375 ms of the inferred neural population firing rates (100 to 475 ms after target onset) were used to predict movement velocities 100 ms later (200 to 575 ms after target onset). Shallow feedforward neural networks were trained to predict velocity from population firing rates. The networks have two hidden layers of 12 and 8 units, respectively, with nonlinear activation functions. The two output layer units, for x and y velocities, are linear combinations of the outputs of the second hidden layer units. Decoded movement velocities of individual trials were obtained using these neural network decoders with 10-fold cross-validation. The angular error between true and decoded movement velocity was then calculated for acceleration and deceleration phases (two intervals before and after peak velocity) of each reach.

### Recurrent neural network

We trained recurrent neural networks (RNNs) to replicate neural activity recorded from the primary motor cortex during reaching movements. Each of the N(=1,000) units in the RNN behaves following a differential equation of the form

(2) $$\tau {\dot{x}}_{i}(t)=-x_{i}(t)+\sum_{j=1}^{N}W_{ij}^{rec}\;r_{j}(t)+\sum_{j=1}^{K}W_{ij}^{in}\;u_{j}(t)+b_{i}$$

where $x_{i}$ denotes the activation of unit i and $r_{i}$ its firing rate; $u_{j}$ represents the j-th extrinsic input; $W_{ij}^{rec}$ is the recurrent connection weight from unit j to i, and $W_{ij}^{in}$ is the weight from input j to unit i. Each unit has a bias, $b_{i}$. A single time constant τ is used for all units in the network.

Extrinsic inputs, u(t), were created based on the rPCA components of the recorded motor cortical neural activity during reaching. There were three groups of extrinsic inputs, corresponding to the three pairs of rPCA components (e.g., Fig. 1D). Each rPCA component was fit with a Gaussian kernel to find the timing of the peak ($\mu_{i},i=1,2,3)$ and the width (represented by its standard deviation $\sigma_{i}$). The temporal offsets between peaks of rPCA components and the three behavioral landmarks were then calculated as $\Delta t_{i}=\mu_{i}-t_{i}$, where $t_{i},i=1,2,3$ represent trial-averaged timing of movement onset, maximum speed, and movement offset, respectively. Note that rPCA analysis and the behavioral landmarks $\left(t_{i}\right)$ were based on data temporally aligned across trials (see Data pre-processing subsection above).

When creating extrinsic inputs for RNN in individual trials, the first extrinsic input group followed

(3) $$u_{j}^{l}\left(t\right)=0.5+0.5*\text{cos}\left(\theta^{l}-\theta_{j}^{PD}\right)*\text{exp}(-{\left(t-\left(\Delta t_{1}+t_{1}^{l}\right)\right)}^{2}/\left(2\sigma_{1}^{2}\right)$$

where $\theta^{l}$ represents target direction in trial $l,\theta_{j}^{PD}$ is the preferred direction of the j-th input (j=1,2,…,18), and $t_{1}^{l}$ is the time of movement onset in trial l. The remaining two input groups were similarly defined using timing of maximum speed and movement offset, respectively. There are 18 inputs in each of the three input groups (therefore K=54), with preferred directions distributed evenly around a circle at 20-degree intervals.

The activation, $x_{i}(t)$, of a recurrent unit was converted to firing rate via the rectified hyperbolic tangent function,

$$r_{i}(t)=\left\{\begin{array}{cc} 0 & \text{if}\;x_{i}(t)\leq 0;\\ \text{tanh}\left(x_{i}(t)\right) & \text{otherwise.} \end{array}\right.$$

The RNN has M output units, which are made identical to a subset of M randomly selected recurrent units of the network (RNN diagram in Fig. 4, nodes in black). Networks were optimized such that the activities of the M output units match firing rates of recorded motor cortical neurons. Training of RNNs aimed to minimize the cost function

$$J=E+\alpha R_{L2}+\beta R_{FR}+\gamma R_{J}$$

where E is the squared error between network output and recorded neuronal activity; $R_{L2}$ is a standard L2 penalty on the input weights and recurrent weights; $R_{FR}$ helps prevent permanent saturation of RNN units; $R_{J}$ encourages simple state-space trajectories. Hyperparameters α(=1e-5),β(=1e-3) and γ(=1e-5) are weights of regularization terms. Detailed explanation of the RNN model can be found in Sussillo et al. 2015^55^.

All single-trial data were randomly divided into five folds. The RNN model was trained with four folds of data and then tested on the remaining fold. This training was repeated five times such that each fold of data acted as the test dataset once, i.e., five-fold cross validation. Training of RNN models was performed using Bridges-2^106^ at Pittsburgh Supercomputing Center.

### Extrinsic and Intrinsic Contributions to RNN Units

After the RNN was trained, we calculated the contributions to the change of activation of individual RNN units. According to Eq. 2, contributions to ${\dot{x}}_{i}(t)$ come from two sources, the external inputs and the intrinsic recurrent network structure. The mean contribution, during a temporal epoch between $t_{1}$ and $t_{2}$, from external input j to RNN unit i was calculated following Eq. 4.


(4) $$C_{i,j}^{ext}=\frac{1}{t_{2}-t_{1}}\int_{t_{1}}^{t_{2}}W_{ij}^{in}\;u_{j}(t)dt$$

Repeating this analysis for all 16 movement directions and all 54 external inputs yielded a 16×54 matrix. This matrix was then split into three 16×18 matrices, each corresponding to one of the three extrinsic input groups. These matrices were visualized using heatmaps and are shown as one row of the left panel in Fig. 4 (Extrinsic Contributions). This analysis was performed in three temporal epochs (early, middle, and late) during the trial. Each epoch was 120 ms long, with its midpoint aligned to the peak of the corresponding group of bell-shaped inputs (see Eq. 3). The same epochs were also used when analyzing the SNN model (see Fig. 6).

To compute the contributions from intrinsic network structure, all RNN units were divided into 18 groups according to their preferred directions during the epoch. The combined contribution from the k-th RNN unit group, $G_{k}$ was calculated using Eq. 5.


(5) $$C_{i,k}^{int}=\sum_{j\in G_{k}}\frac{1}{t_{2}-t_{1}}\int_{t_{1}}^{t_{2}}\left(W_{ij}^{rec}\;r_{j}(t)-\frac{x_{i}(t)}{N}+\frac{b_{i}}{N}\right)dt$$

The contributions of 18 RNN unit groups for 16 movement directions during each of the three temporal epochs were presented as heatmaps in Fig. 4 (Intrinsic Contributions).

### Directionality test

The differential extrinsic and intrinsic contributions to the directional tuning of an RNN unit were calculated using the correlation coefficients between the tuning curve of the RNN unit and those of extrinsic and intrinsic contributions, respectively. To get the tuning curve of the intrinsic contributions, the values in a 16×18 heatmap (described in Extrinsic and Intrinsic Contributions to RNN Units, above) for a given temporal epoch were summed across all columns, resulting in a 16×1 vector representing the total contributions for the 16 movement directions from all RNN units. Similarly, the tuning curve for extrinsic inputs, a second 16×1 vector, was found by summing the 54 columns of the three 16×18 matrices of contributions from the three input groups. The mean firing rate of the target RNN unit in that epoch was also calculated for all movement directions, forming a third 16×1 vector. The correlation coefficient between the vector of contributions and the firing rate vector was then calculated for extrinsic and intrinsic contributions, respectively. This analysis was repeated for all three temporal epochs and for all RNN output units. The distribution of these correlation coefficients across all output units in each epoch is shown as a histogram in Supplementary Fig. S9. This analysis reveals that contributions from external inputs had stronger correlation with output firing rates than those from intrinsic drivers.

### Dropout test

For the input dropout test (Supplementary Fig. S10), the latter two groups of extrinsic inputs were set to a constant value of 0.5 for all times. This test shows how RNN outputs would change after losing some of the extrinsic inputs.

### RNN state space trajectory

The state-space trajectories of the model were visualized by performing rPCA on the activations of all RNN units, $x(t)={\left[x_{1}(t),\ldots ,x_{N}(t)\right]}^{T}$. A 3-dimensional state space was formed using three of the top six rPCA scores, one from each of the three pairs of components. Single-trial x(t) was then projected into this 3-D space to visualize the state-space trajectory (Fig. 3C). The gradient, $\dot{x}(t)$, was then calculated along the trajectory. Similar to the contribution analysis above, we split the gradient into two parts as driven by extrinsic inputs and intrinsic network structure, respectively. These two parts of $\dot{x}(t)$ were then projected back to the above 3-D space and plotted as arrows in Fig. 3C (green arrows for extrinsic inputs and purple for intrinsic dynamics). A 2-D flow field within this 3-D space was made to further visualize the intrinsic RNN dynamics. Evenly spaced sample points ($\left[s_{1},s_{2}\right]$ value pairs) were taken from this 2-D plane. The averaged values across time and trials of the remaining N-2 dimensions were calculated (a vector of $\left[s_{3},\ldots ,s_{N}\right]$, and then combined with each of the above 2-D sample points, respectively, to form N-D vectors. These vectors were then projected back to the original N-D space using rPCA axes to find the corresponding $x={\left[x_{1},\ldots ,x_{N}\right]}^{T}$ vectors. Using Eq. 2 above, we calculated $\dot{x}$ for each x, and then split it into two parts, ${\dot{x}}^{\text{int}}$ and ${\dot{x}}^{\text{ext}}$, corresponding to the intrinsic network structure and extrinsic inputs, respectively. The ${\dot{x}}^{\text{int}}$ vector for each sample point was projected back to the previous 2-D plane and plotted as a purple arrow for each sample point on the bottom plane of Fig. 3C.

### Spiking neural network

The spiking neural network (SNN) was constructed to replicate recorded neural activity from a set of simulated extrinsic inputs. The network was constructed using four groups of 90 neurons that were fully connected to the output neurons (Supplementary Fig. S11A). Following the same motivation as that for the RNN, the network was trained so that the firing rates of the output neurons matched those of the neurons recorded empirically during reaching.

The input comprised three sets of 90 neurons with directional tuning during the epochs identified by rPCA, and one non-directional set of 90 neurons tuned to reach speed. The Brian2 simulator^58^ was used to generate input spikes from an underlying drive function. For the directional inputs, the drive was modeled as a Gaussian fit to the modulation of the rPCA data (as described above in the RNN section), scaled by the cosine of each neuron’s preferred direction, which was evenly distributed around a circle. These profiles were further scaled by movement speed and included a positive offset to increase the baseline firing rate, with additional noise to reduce correlated firing between input units. The peaks of the profiles in each input group were aligned to the behavioral landmark in its corresponding epoch in each trial. For the non-directional group, the drive followed the hand’s speed profile, shifted backward by 50 ms, with neurons having a gain distributed from −0.5 to 1.0, plus an offset and noise.

The weights were trained using the relative timing of input and output (recorded) neuron spikes. The potential contribution to weights for each input neuron was tracked individually. The baseline contribution is negative and at the time of each spike, a two-sided decaying exponential was added onto the baseline. The value of the potential contribution was added to the weight between the input and output unit at the time of the output unit spiking. If input and output neuron’s spikes coincided, a positive contribution was added to the weights, otherwise a negative contribution was added. The baseline contribution was adjusted so the average weight into an output unit from an input group was zero (Supplementary Fig. S11B).

The network was optimized by scaling the weights from each input group into each output and by adjusting the modeled output unit thresholds to minimize the RMSE between predicted and actual firing rates. A differential evolution optimization method^107^ was used to iteratively evaluate parameter combinations and combine the best performers for the subsequent iteration evaluation.

Finally, the network was simulated for all trials using the optimized weights and thresholds. The membrane potential for each output unit was tracked, with changes occurring whenever an input unit fired, influenced by the connecting weight. The membrane potential also tended towards a resting potential.

### Statistical structure of synaptic integration

Analyses were performed to explore the statistical structure of input spikes leading to output spikes in the SNN model. Because of the deterministic quality of the SNN model, the precise effect on output neuron membrane potential from each input spike can be quantified. To find which input spikes lead to an output spike during each of the three epochs, we restricted analyses to three distinct segments of the reach (Fig. 6), as described in the main text. Input spikes occurring within a 20 ms (*buildup*) or 0.1 ms (*trigger*) window before output spikes in these segments were considered for analysis.

First, raw spike counts were extracted. There were 360 individual input units (90 units × 4 input groups), 16 target directions, and three epoch segments (Fig. 6). Input spikes occurring in either window before an output spike were counted. To get spike probability, the spike counts within each target/epoch combination were divided by the total number of input spikes occurring in the target/epoch. Finally, to get the weighted contribution to membrane potential as shown in Fig. 6, the spike probability was multiplied by the input’s corresponding weight. These analyses were completed for both the buildup and trigger windows.

An additional analysis used with the weighted contributions. Fig. 6 shows a strong diagonal, which indicates input units with a preferred direction that matches the direction of movement have relatively large contributions to the output unit membrane potential. We quantified this phenomenon using the directionality test (below) for all units in our data set.

### Diagonal test

We down-sampled the 90 columns of the heatmaps to 16 in order to create a square (16×16) matrix and then calculated a diagonality metric, which is simply the sum of the magnitudes of contributions along the diagonal divided by the sum of all 256 contribution magnitudes. This metric was calculated for all output units, epoch segments, and the three directional input groups for the *buildup* window. A bootstrap analysis was performed on each matrix by randomly selecting 16 of 256 contributions in the matrix (without resampling each row or column) and calculating the diagonality metric using these contributions as the “diagonal.” The selection was repeated 1000 times and compared these randomly generated diagonality metrics to the actual diagonality metrics. Epoch segments that did not contain at least one output spike in all movement directions were dropped. An output unit was removed if all three epochs were dropped, leaving 62/67 units for Monkey C and 58/78 units for Monkey N. All the actual diagonality metrics were greater than the corresponding randomly generated diagonality metrics.

To test whether positive and negative contributions to the membrane potential along the diagonal balanced, the values of the diagonal were regressed against a cosine function. For both Monkeys C and N, all remaining units had an input group/epoch combination with an ${\text{R}}^{2}>0.9$. The cosine functions for all units were approximately centered on 0, showing that the positive and negative contributions were balanced.

## Supplementary Files

This is a list of supplementary files associated with this preprint. Click to download.

- supplementalfiguresNatureNeuroscience.pdf
